# Molecular farming for sustainable production of clinical‐grade antimicrobial peptides

**DOI:** 10.1111/pbi.14344

**Published:** 2024-04-29

**Authors:** Shahid Chaudhary, Zahir Ali, Magdy Mahfouz

**Affiliations:** ^1^ Laboratory for Genome Engineering and Synthetic Biology, Division of Biological Sciences 4700 King Abdullah University of Science and Technology Thuwal Saudi Arabia

**Keywords:** antimicrobial peptides, molecular farming, green synthesis, clinical‐grade AMP production

## Abstract

Antimicrobial peptides (AMPs) are emerging as next‐generation therapeutics due to their broad‐spectrum activity against drug‐resistant bacterial strains and their ability to eradicate biofilms, modulate immune responses, exert anti‐inflammatory effects and improve disease management. They are produced through solid‐phase peptide synthesis or in bacterial or yeast cells. Molecular farming, i.e. the production of biologics in plants, offers a low‐cost, non‐toxic, scalable and simple alternative platform to produce AMPs at a sustainable cost. In this review, we discuss the advantages of molecular farming for producing clinical‐grade AMPs, advances in expression and purification systems and the cost advantage for industrial‐scale production. We further review how ‘green’ production is filling the sustainability gap, streamlining patent and regulatory approvals and enabling successful clinical translations that demonstrate the future potential of AMPs produced by molecular farming. Finally, we discuss the regulatory challenges that need to be addressed to fully realize the potential of molecular farming‐based AMP production for therapeutics.

## Introduction

**Figure 1:**
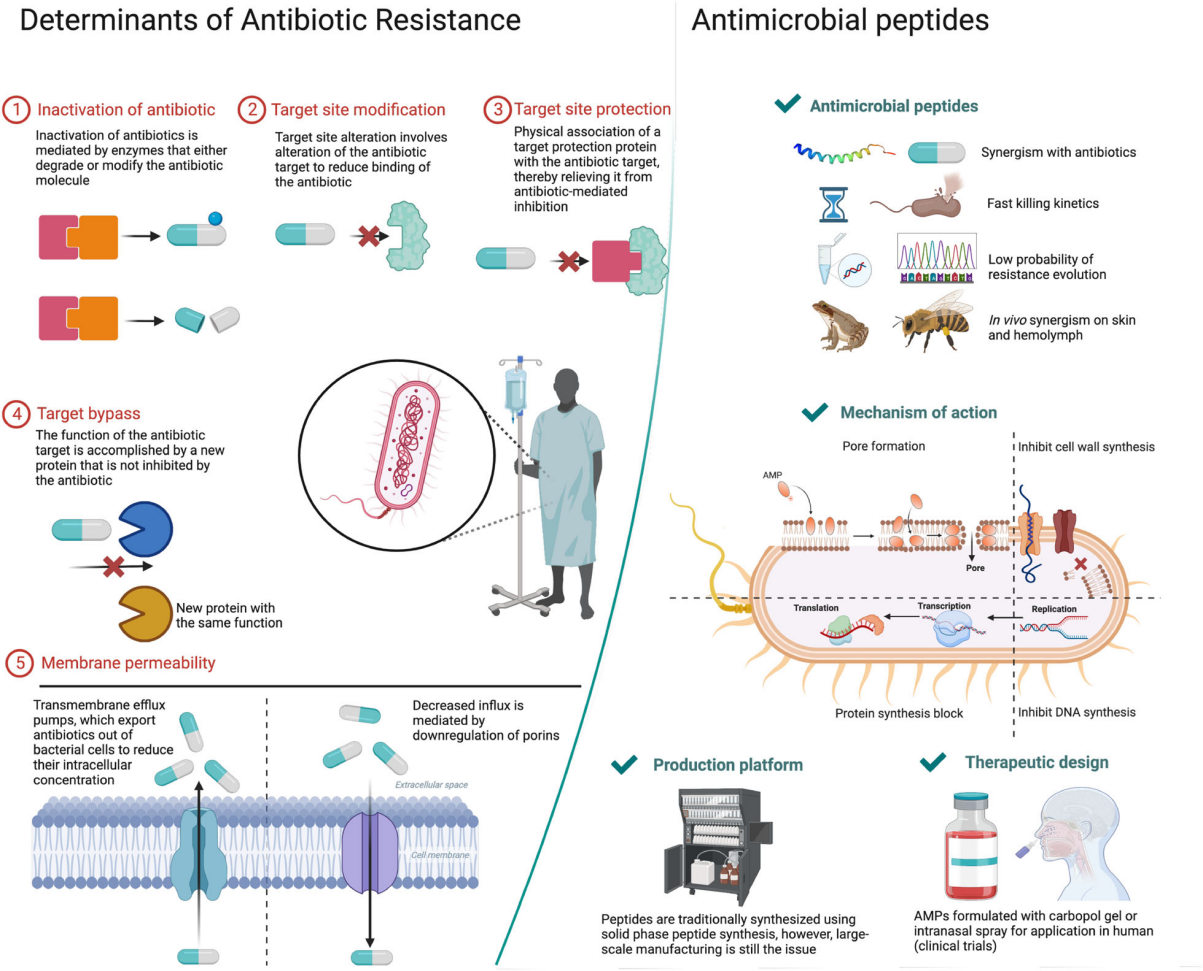
Schematic representation of the mechanisms of antibiotic resistance and AMPs as promising approaches to combat resistant infections. Against the backdrop of increasing antimicrobial resistance, AMPs are emerging as an alternative antibiotic. The pharmacodynamics of AMPs are more favourable compared to the conventional antibiotics, and bacteria exhibit a lower propensity to develop resistance in comparison to antibiotics (Jochumsen *et al*., 2016; Kaufmann *et al*., 2021; Kintses *et al*., 2019; Rodriguez‐Rojas *et al*., 2014). Besides, AMPs show rapid killing kinetic (Fantner *et al*., 2010) through its stereotypical mechanism of creating pores in the cell wall, while other intracellular targets are also involved. Another important aspect of AMPs from a clinical perspective is their potential to be combined with existing antibiotics that have lost effectiveness due to resistance (Lazzaro *et al*., 2020; Lin *et al*., 2015), which raises the possibility of reviving antibiotics and achieving unprecedented potencies that have not been reported before. In context to produce AMPs for clinical application, SPPS standout as the primary method, and recombinant techniques can also be applied for large‐scale production.

Driven by the global emergence of antibiotic resistance and the lack of new antimicrobial drugs in drug development pipelines, the discovery of antimicrobial peptides (AMPs) has been touted as a promising solution to this pressing medical crisis (Kuo *et al*., 2013). AMPs are short ∼10 to 60 amino acid (aa) peptides (in contrast to antimicrobial proteins of more than >60 aa) and occur in plants, animals and fungi. For example, cationic host defence peptides (HDPs, 45–54 aa) such as defensins are found in animals and plants, have microbicidal properties (Huan *et al*., 2020) and interact with the host immune system to combat infections (Mookherjee *et al*., 2020; Figure 1).

**Figure 2:**
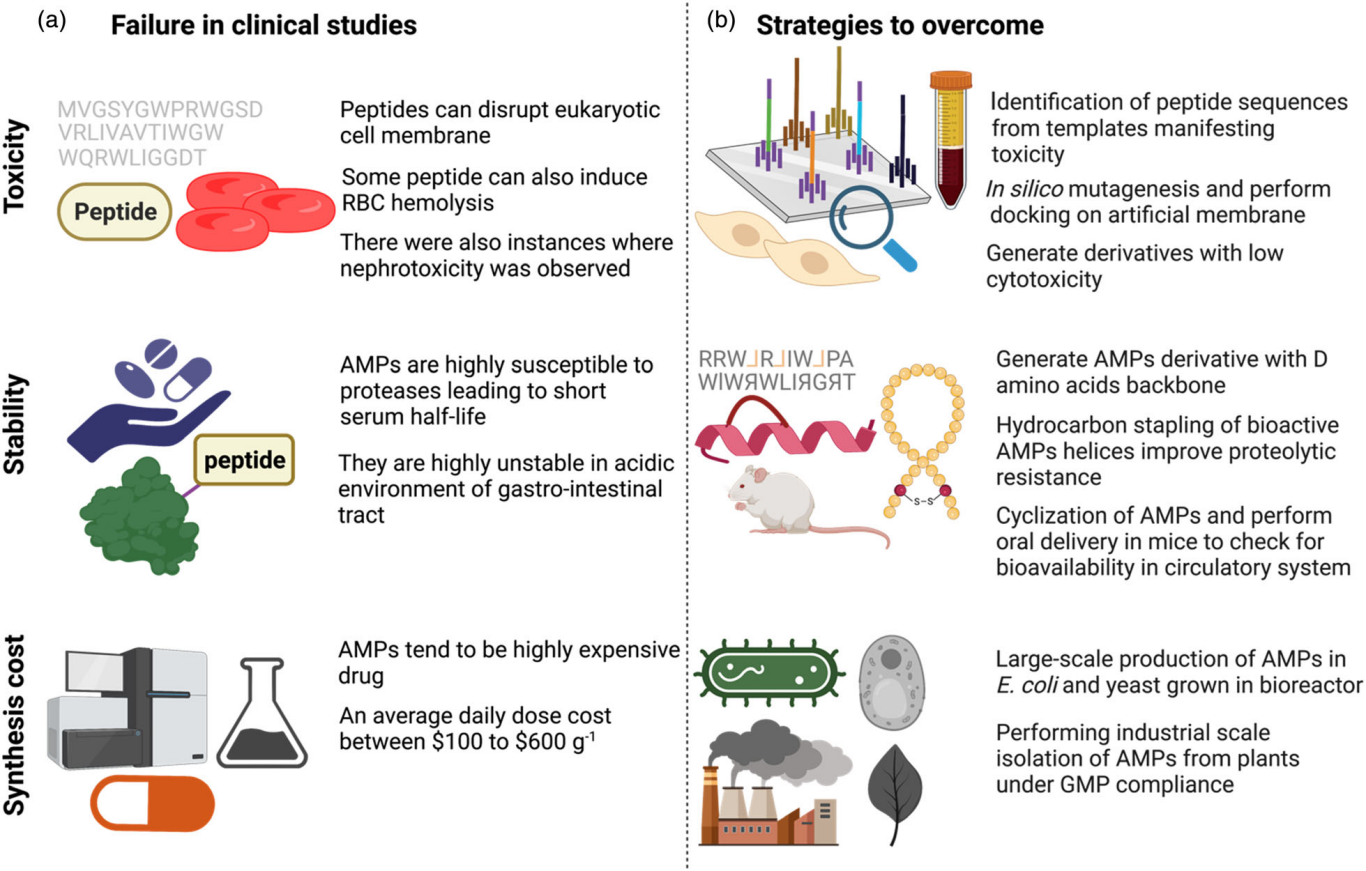
Enhancing AMPs' clinical potential. (a) Overview of the failure of AMPs in clinical studies, including toxicity towards host cells, the rapid degradation of peptides in serum reducing their serum retention time and the high production cost associated with their manufacturing. (b) Possible strategies to improve the pharmacokinetic/pharmacodynamic properties of AMPs to achieve the same clinical outcomes as conventional antibiotics.

Although AMPs hold significant promise, their clinical translation remains limited due to insufficient drug development and the lack of a robust drug manufacturing platform (Figure 2; Fox, 2013), in addition to challenges related to unfavourable pharmacokinetics and potential toxicity (Figure 2; Hancock and Sahl, 2006). Traditionally, short peptides (<50 aa) are synthesized using solid‐phase peptide synthesis (SPPS), but this process requires extensive utilization of hazardous chemicals, raising concerns related to environmental effects, sustainability and cost (Isidro‐Llobet *et al*., 2019). Additionally, recombinant systems, such as insect cell lines, bacterial cultures, yeast cultures and cell‐free extracts, are being optimized to improve yield to meet demands for AMPs in medical applications. As an alternative, ‘green’ solutions using the biological engineering of plants for AMP synthesis offer the potential for low‐cost, high‐yielding, scalable and environmentally safe approaches to bridge the sustainability gap.

The biosynthesis and biotechnological production of AMPs in plants and other host organisms have been comprehensively discussed in previous articles (Holaskova *et al*., 2015; Parachin *et al*., 2012). This review discusses the technical advantages of molecular farming for the production of clinical‐grade AMPs, along with relevant updated advances in peptide expression and purification systems; provides a techno‐economic assessment analysis of the use of molecular farming for industrial‐scale production and describes how molecular farming can bridge the gap in environmental sustainability, cultural acceptability and low‐cost accessibility, as well as streamlining patent grants, clinical translation and the U.S. Food and Drug Administration (FDA) approvals. We have also updated recent advancement in utilizing *Escherichia coli* and yeast systems for AMP production, with the latter leading to clinical application. Finally, we discuss the regulatory challenges that need to be addressed to take full advantage of molecular farming‐produced AMPs for therapeutic purposes.

## Antimicrobial peptides

Antimicrobial peptides have demonstrated rapid evolution across various species and kingdoms, adapting to the distinct challenges posed by pathogenic microbes in different host organisms over time. Their simple biochemical properties, as described below, facilitate the *de novo* evolution of AMPs, and certain three‐dimensional architectures have independently evolved across diverse taxa, including plants, insects and vertebrates (Broekaert *et al*., 1995; Shafee *et al*., 2017). Defensins, an extensive superfamily of AMP, are ubiquitously evolved across various taxa but display substantial variations among species. For instance, mice lack neutrophil α‐defensins, the most prevalent proteins in human neutrophils, while cattle completely lack α‐defensins in both the gut and neutrophils (Patil *et al*., 2004). Similarly, the cathelicidin peptides, mouse cathelin‐related AMP (mCRAMP) and human LL‐37 exhibit only a 67% homology, and neither species possesses the substantial diversity of cathelicidins found in cattle fields (Patil *et al*., 2004). It seems plausible that the rapid co‐evolution of host‐defence peptides and microbial counter strategies, including resistance and virulence strategies, is likely a key driver of diversity across all biological kingdoms (Bowdish *et al*., 2005; Peschel and Sahl, 2006).

Given that the majority of antibiotics developed in modern medicine have their origins in microbial sources, it is reasonable to speculate that bacteria may also naturally possess AMPs to defend against predators. Nisin, the first AMP isolated from the bacterial strain *Streptococcus lactis*, imparts a cytotoxic effect on other types of bacteria, allowing it to compete for nutrients in the environment (Mattick and Hirsch, 1947). Peptide AN5‐1 (YSKSLPLSVLNP) was originally reported in the fermentation broth of *Paenibacillus alvei* strain AN5 (Alkotaini *et al*., 2013). It has multiple cellular targets in bacteria, including damaging the bacterial membrane and binding to DNA to disrupt cellular functions (Yi *et al*., 2015). Moreover, a recent study indicates that the intestinal microbiota acts as a reservoir for AMPs, which include bacteriocins (Garcia‐Gutierrez *et al*., 2019; Pushpanathan *et al*., 2019). Typically, these bacteria are inherently resistant to the AMPs they produce, implying an evolutionary adaptation of peptides within bacteria to protect themselves in their environment.

### Structure of AMPs

AMPs have relatively simple structures, commonly exhibiting α‐helical and β‐like structures (Torres *et al*., 2022a). A small subset of natural AMPs form extended/random‐coil peptides, lacking a defined structure, and are often rich in arginine, proline, tryptophan and/or histidine residues (Nguyen *et al*., 2011; Takahashi *et al*., 2010). Most AMPs are α‐helical, since most are cationic and amphipathic; from a peptide design perspective, any notable changes to this α‐helix resulting from amino acid substitutions or stapling (Mourtada *et al*., 2019) substantially affect AMP antimicrobial efficacy (Lee *et al*., 2016).

AMPs with β‐sheet structures are associated with robust stability and activity (Akishiba *et al*., 2017; Fan *et al*., 2020). The β‐sheet structural configuration allows the AMPs to form supramolecular assemblies, creating pores that can penetrate host membranes. The ability to penetrate membranes allows β‐sheet AMPs to interact with intracellular targets, including promoters, mRNA‐binding motifs, enzyme regulatory regions and protein‐folding domains, resulting in inhibitory effects and eventual cell death in pathogenic microorganisms (Sharma *et al*., 2016).

### Charge

The majority of AMPs (Nazarian‐Firouzabadi *et al*., 2023) and microbicidal proteins (Shang and Nelson, 2019; Walker *et al*., 2007; Yamauchi *et al*., 1993) are cationic, which allows them to electrostatically interact with the negatively charged phospholipids of the bacterial membrane (Haney and Hancock, 2013; Haney et al., 2019). Anionic AMPs are also found in nature, primarily arising from peptide fragmentation due to proteolysis; however, some anionic AMPs also exist as small molecules encoded by genes (Dennison *et al*., 2018).

Numerous studies have shown a strong correlation between cationicity and defence activity of AMPs (Bessalle *et al*., 1992; Dathe *et al*., 1996; Matsuzaki *et al*., 1996). Naturally occurring AMPs have 0 to more than 20 positive charges, with the most effective peptides typically having a net charge between +3 and +6 (Haney and Hancock, 2013). However, the killing sensitivity of peptides is not solely defined by their charge, as additional factors, such as secondary interactions, solvation and amino acid composition, also dictate how these molecules interact and potentiate broad‐spectrum activity.

### Hydrophobicity

The bacterial membrane consists primarily of lipids, and consequently, it is unsurprising that hydrophobicity must be integrated into the AMP backbone for antimicrobial efficacy. The proportion of hydrophobic residues in naturally occurring peptides typically ranges between 40% and 60%, and this aligns with the requirement for energetically stable amphipathic structures crucial for antimicrobial function (Tossi *et al*., 2000). The hydrophobicity of AMPs (magainin, melittin) and proteins, including the proteins lactoferrin (Nakamura *et al*., 2022) and lysozyme (Pal and Mitra, 2022), are crucial for their efficacy against microbes, as these antimicrobials leverage their hydrophobic region to disrupt bacterial membranes, ultimately leading to lysis of the cell. However, excessive hydrophobicity might trigger peptide aggregation or nonspecific interactions with host cells. Therefore, researchers continue to explore modifications of AMP hydrophobicity, aiming to enhance their potency against pathogens while mitigating potential cytotoxicity to host cells.

### Amphipathicity

The amphipathicity of peptides, including the proteins colicin (Bohme *et al*., 2009), lactoferrin (Sinha *et al*., 2013) and lysozyme (During *et al*., 1999), directly influences their mechanism of action and thus their antimicrobial activity. Amphipathicity is often correlated with peptide helicity; however, it is important to note that amphipathic properties are not confined to helical structures, as β‐turns or β‐sheets, can also exhibit substantial amphipathicity depending on their sequence (Jin *et al*., 2005). These amphipathic molecules are characterized by a charge, often cationic, and multiple hydrophobic moieties. The cationic moiety is responsible for the electrostatic interaction with the peptide–membrane interface, while the hydrophobic regions directly interact with the hydrocarbon chains in the lipids. Amphipathicity is closely linked to the hydrophobic moment of the molecule, which is directly influenced by the specific secondary structure of the peptide, as elucidated in the Eisenberg plot (Keller, 2011).

### Mechanism of action

AMPs have two general modes of action: targeting membranes and targeting intracellular components. Many AMPs, such as melittin and defensins, interact with bacterial cell membranes through electrostatic attractions. Several models have been developed to explain how AMPs interact with membranes, such as the toroidal pore model (Wimley, 2010), the carpet model (Han *et al*., 2017) and the barrel‐stave model (Shabir *et al*., 2018). Our understanding of the mechanisms by which AMPs interact with membranes remains incomplete due to the obscure nature of nonspecific interactions, impeding accurate prediction of the optimal physicochemical balance required for distinct membrane‐targeting actions. However, some microbicidal proteins, such as colistin and collectins, have a distinct binding affinity for lipopolysaccharide or conserved carbohydrate moieties, respectively, on the outer membrane of bacterial cells (Murugaiah *et al*., 2020; Sabnis *et al*., 2021). This specific binding impairs the integrity of the cell membrane, eventually leading to cell death, or helps foster a robust immune response.

In addition to targeting membranes, certain AMPs and proteins also affect membrane‐associated processes such as cell wall biosynthesis and cell division, or cytoplasmic processes, including macromolecular synthesis and heat shock proteins (Fjell *et al*., 2011; Hale and Hancock, 2007; Otvos Jr., 2005). For example, Buforin II, an AMP derived from histones of Asian toads (*Bufo bufo gargarizans*), enters the bacterial cytoplasm and binds to DNA and RNA (Park *et al*., 1998). Similarly, colicin proteins (E2–E9) have nuclease activity; moreover, colicin M inhibits cell wall biosynthesis via enzymatic degradation of undecaprenyl phosphate‐linked peptidoglycan (murein) precursors (Cascales *et al*., 2007).

## Conventional AMP production systems

Currently, the manufacturing of AMPs is dominated by SPPS‐based synthesis, which is compatible with synthesizing complex peptides, including those requiring the incorporation of non‐canonical amino acids (Der Torossian Torres and de la Fuente‐Nunez, 2019). Although SPPS methods for synthesizing peptides are relatively straightforward, there is no direct correlation between synthesizing peptides in small quantities (1–10 mg) and at a large scale (g to kg).

In SPSS, the final output of the synthesized peptide is strongly influenced by the nature of the amino acid sequences added to the AMP backbone. Peptides containing a stretch of hydrophobic residues frequently aggregate during synthesis, forming insoluble particles that are challenging to separate from the reaction mixture (Ajikumar and Devaky, 2001). The SPPS platform has notable limitations in synthesizing long peptides (>50 aa) (Raibaut *et al*., 2015), as the effectiveness of coupling and de‐protection steps radically decreases, primarily due to the sequence‐dependent propensity of longer peptides to aggregate (Winkler and Tian, 2015). Furthermore, the chemicals utilized for coupling and de‐protection can be hazardous, and SPSS therefore generates significant amounts of waste solvents (Isidro‐Llobet *et al*., 2019). Therefore, for the sake of sustainability, scalability and cost‐effective production of peptide drugs, the U.S. FDA promotes their synthesis in biological systems, such as bacteria and yeasts, to create alternative, efficient and cost‐effective platforms for large‐scale peptide production.

Bacterial systems, in particular *E. coli*, have been extensively used to express AMPs (Overton, 2014), due to their ease of culture and rapid growth (Owczarek *et al*., 2019). Moreover, growth of *E. coli* in Notomista–Arciello broth achieved an unprecedented increases in cell biomass with concomitant production of HDPs, thereby decreasing production cost of the frog (*Rana pipiens*) onconase AMPs ONC‐r(P)GKY20, ONC‐r(P)ApoBL and ONC‐r(P)ApoBS from €253 to €42 mg^−1^ (Gaglione *et al*., 2019). The inherent toxicity of AMPs towards bacterial cells can be overcome by a fusion strategy in which the peptide sequence is masked to reduce its toxicity to the host cell. For example, Ishida *et al*. (2016) created AMPs fused to the eukaryotic calcium sensor protein calmodulin (Ishida *et al*., 2016).

*Escherichia coli* can be used for the bulk production of clinical‐grade AMPs, which modulate the immune response (IDR‐1) or suppress inflammation (MX‐226) (Bommarius *et al*., 2010). However, bacterially produced peptides are not amidated, unlike their synthetic counterparts, which are currently being evaluated in clinical trials. To address this shortcoming, Unigene Laboratories (U.S.) developed a new method of producing amidated peptides with high efficacy in genetically engineered *E. coli* cells (European patent, EP2088188A1). To further improve AMP pharmacokinetics, researchers are currently altering bacterial ribosomes to incorporate non‐canonical amino acids (Reitz *et al*., 2018), a process that can potentially be used for large‐scale production of AMPs with enhanced therapeutic index.

In addition to their clinical applications, bacterially made peptides have been used as a preservative in the dairy and meat industries (Magana *et al*., 2020). Nisin, an AMP approved for use in over 48 countries, is purified commercially from *S. lactis* (U.S. patent no. US2935503‐A) but remains costly to produce. In addition to these AMPs, many antimicrobial proteins have been successfully expressed in *E. coli* (Table 1).

**Table 1:** List of antimicrobial proteins produced using *Escherichia coli*, yeast, cell‐free extracts and plant‐based platforms

Chassis used to produce protein	Antimicrobial protein	Pathogen(s) targeted	References
*E. coli*	Colicin M, Lysep, MS2, SRRz, X174E	*E. coli*	Diao *et al*. (2021)
	Colicin F	*Yersinia enterocolitica*	Bosak *et al*. (2018)
	Salmocin M	*Salmonella* Enteritidis, *S*. Paratyphi, *S*. Typhimurium, *S*. Gallinarum, *S*. Hadar, *S*. Virchow	Lojewska *et al*. (2022)
	Endolysin TP84_28	*Bacillus cereus, B. subtilis, E. coli*	Zebrowska *et al*. (2022)
	Endolysin PlyGRCS	*Staphylococcus aureus, S. epidermidis*	US patent US9872893B2
	Pyocin AvR2‐V10.3	*E. coli* O157:H7	Ritchie *et al*. (2011)
	Chimeric Enterocin A‐colicin E1	*S. aureus*	Fathizadeh *et al*. (2020)
Yeast	Hen egg white lysozyme	*Micrococcus lysodeikticus*	Cui *et al*. (2022)
	Endolysin LysSA11	*S. aureus*	Chun *et al*. (2020)
	Endolysin PVP‐SE1gp146	*Pseudomonas aeruginosa, E. coli, and Acinetobacter baumannii*	Asadi‐Saghandi *et al*. (2023)
Cell‐free extract	Colicins M, Ia, E1 and E2	*E. coli* strain K361	Jin *et al*. (2018)
Plant	Pyocins S5, PaeM, L1, L2, L3 and PaeM4	*P. aeruginosa*	Paskevicius *et al*. (2017))
	Colicins M, K, U and 1b	*E. coli* strain DH10B	Stephan *et al*. (2017)
	Salmocins SalE1a and SalE1b	*S*. pathovars strains	Schneider *et al*. (2018)
	Lysins psm, ZP173, PlyCP26F, PlyCP39O and CP25L	*Clostridium perfringens* strains	Kazanaviciute *et al*. (2018)
	Klebicins KpneM, KpneM2, KvarM, KaerM	*Klebsiella pneumoniae, K. quasipneumoniae, K. oxytoca, K. variicola and K. aerogenes*	Denkovskiene *et al*. (2019)
	Chimeric bacteriocin S5‐PmnH	*P. aeruginosa*	Paskevicius *et al*. (2022)

Lysep, *E. coli* bacteriophage lysin; MS2, lysin from bacteriophage MS2; SRRz, lambdoid lysis cassette; TP84, bacteriophage; PlyGRCS, the *S. aureus*‐specific bacteriophage endolysin; AvR2‐V10.3, tail spike from bacteriophage phiV10 is fused to the R2 pyocin tail fibre; LysSA11; *S. aureus* phage SA11 lysin; PVP‐SE1gp146, derived from the bacteriophage PVP‐SE1 specific to *Salmonella* Enteritidis; PaeM, pyocin M1; PlyCP, endolysin of *C. perfringens* bacteriophage.

Like bacteria, yeast cells represent promising routes for AMP biomanufacturing, as they are immune to AMP‐mediated toxicity and amenable to post‐translational modifications that closely resemble those in human cells (Torres *et al*., 2021). In particular, the yeast *Pichia pastoris* has been used to produce various prototypical AMPs that it secretes into its growth medium, facilitating continuous AMP production at a reduced cost for purification (Basanta *et al*., 2010). *Pichia pastoris* has a well‐regulated and tightly controlled inducible expression system, eliminating the need for carrier proteins in the production of defensins (Wang *et al*., 2009; Zhao and Cao, 2012), plectasin‐derived peptides (Mao *et al*., 2015; Zhang *et al*., 2014) and cecropin (Guo *et al*., 2012; Wang *et al*., 2011). A study used genetically engineered *P. pastoris* for the production of recombinant AMPs in bioreactors produced 1 g of apidaecin Ia (proline‐rich peptide from *Apis mellifera*), a yield much higher than those obtained in *E. coli*, at a maximum cost of US$1 g^−1^ (Cao *et al*., 2018). Pezadeftide (a plant defensin) produced using a *P. pastoris* platform (Hexima Limited, https://hexima.com.au; Australia) was translated to phase 2 trials for topical treatment of the fungal infection onychomycosis. However, the cost associated with scaling up the peptide production exceeded initial estimates by 75%, and furthermore, the clinical data did not meet the endpoints, which led to the discontinuation of further trials. The yeast platform has also been used to produce antimicrobial proteins (Table 1).

Insect cells (Sf9 and Sf2 cell lines from the moth *Spodoptera frugiperda*) replicate faster than mammalian cells, allowing quicker protein expression, but the costs are higher than those of bacteria and yeast due to the need for specialized culture media (Owczarek *et al*., 2019). Although this platform can achieve superior peptide production (25 mg/L) compared to bacterial systems (typically 10 mg/L but up to 100 mg/L), the peptides produced are vulnerable to degradation by proteases that are produced by insect cells in response to viral transfection (Ikonomou *et al*., 2003; Zitzmann *et al*., 2017). In addition, certain insects harbour viruses that could pose a risk to human health, so it is important to thoroughly assess insect cell lines before obtaining regulatory approval (Felberbaum, 2015).

Finally, cell‐free expression platforms have been used for decades and make it possible to conduct transcription and translation *in vitro* like in an intact living organism (Silverman *et al*., 2020). Peptides (<50 aa) can be synthesized on demand using lyophilized extracts of *E. coli* simply by hydrating the reaction, enabling production of abiotic, sterile and cold‐chain‐independent peptide in a portable platform at low cost (Pardee *et al*., 2016). In a recent development, artificial intelligence (AI) was integrated with high‐throughput screening for candidate AMPs in a cell‐free protein synthesis pipeline, facilitating a streamlined, inexpensive process for producing and testing bioactive peptides within less than 24 h (Pandi *et al*., 2023). Antimicrobials exceeding 100 aa have also been successfully synthesized using *E. coli*‐based cell‐free protein synthesis (Table 1). Cell‐free platforms also can incorporate non‐canonical amino acids into the peptide template, resulting in modified peptides that resist proteolytic degradation and thus have improved efficacy in vivo. In the past, cell‐free systems have had limited capacity for large‐scale production of peptides or biologics; however, Sutro Biopharma has extensively modified its cell‐free system for process scale‐up and good manufacturing practice (GMP) product requirements (Carlson *et al*., 2012). Ongoing improvements in cell‐free systems will likely enhance the scale of AMP synthesis.

## Molecular farming

Plants can express foreign genes in transgenic and transient expression systems, leading to the successful production of numerous therapeutic compounds. Recombinant expression through transgenesis represents a well‐established strategy, but faces drawbacks in term of production, which takes more than a year, and product yield, which is generally in the range of 0.1%–0.5% of total soluble protein (Lobato Gomez *et al*., 2021).

In contrast to transgenic approaches, transient expression systems expedite the recovery of protein in a few days, making these platforms faster and more scalable (Chung *et al*., 2022; Lobato Gomez *et al*., 2021). The advent of transient expression systems led to a major paradigm shift in molecular farming (Rybicki, 2010). These transient systems involve *Agrobacterium tumefaciens*‐mediated delivery of plant virus‐derived vectors and result in robust expression of the exogenous mRNA and protein production surpassing the levels seen in transgenic plants.

Plant‐based platforms have many advantages, including safety, as there are no replicating human pathogens; simplicity, since production does not require sterile conditions; scalability and rapidity, whether through open‐field cultivation of transgenic plants followed by limited downstream processing (DSP) for direct oral use or transient expression systems for industrial‐scale production within weeks. However, despite these advantages, plant‐based production technology has an unclear regulatory pathway, with only a few molecular farming‐based products reaching the markets as approved therapeutics. This lack of clarity has impeded the translation of molecular farming‐based products to clinical applications (Margolin *et al*., 2020).

## Molecular farming of AMPs

Plants are still considered as an ‘emerging’ platform rather than an alternative for AMPs production, given concerns associated with potential damage to plant cell membranes, and notable production cost, particularly in DSP. However, the recent second wave of molecular farming has integrated synthetic biology for producing genetically engineered vectors that outperform current standards, along with the establishment of DSP steps and framework models to predict economic viability, marking a notable achievement in the field (Chaudhary and Mahfouz, 2024). Moreover, recently, machine learning approaches have been employed to predict the required molecular modifications for overcoming the challenges associated with AMP expression in plant fields (Jaiswal *et al*., 2023).

### AMP production from viral vectors

Many early efforts at plant‐based peptide production resulted in low or undetectable expression of the desired peptide (Company *et al*., 2014; Lee *et al*., 2011; Okamoto *et al*., 1998), likely due to incompatibility between the plant host and the peptides being expressed (Ghidey *et al*., 2020). This was overcome with the production of the *de novo*‐designed 12‐amino acid peptide SP1‐1 (RKKRLKLLKRLL) in first‐generation viral vectors, which are essentially functional wild‐type viruses, namely tobacco mosaic virus (TMV). Expressing SP1‐1 fused to TMV‐coated protein yielded up to 0.025 mg/g (infiltrated leaf biomass) of pure (>90%), biologically active AMP (Zeitler *et al*., 2013). This viral system offers advantages for AMP production, and additional peptides are expected to be synthesized using whole‐virus technology; however, it cannot accommodate larger transgenes.

Removing unnecessary parts of the viral genome produced second‐generation viral vectors, often called ‘deconstructed’ vectors such as magnICON (Marillonnet *et al*., 2004), TRBO (Lindbo, 2007), pEAQ (Sainsbury *et al*., 2009) and geminiviral vectors (Chen *et al*., 2011). These vectors were further modified for improved yields, faster production, reduced processing costs, lower R&D expenses and enhanced biosafety. Nomad Bioscience (Halle, Germany) achieved GMP‐compliant production of antimicrobials in plants, reaching up to 3 g/kg of fresh biomass in just 2 weeks (Hahn‐Lobmann *et al*., 2019). The magnICON platform was successfully used to transiently express biologically active, broad‐spectrum AMP protegrin‐1 (PG‐1) in *Nicotiana tabacum* (Patiño‐Rodríguez *et al*., 2013). Although the yield of the peptide obtained was not reported, the study emphasizes the importance of deconstructed vectors for rapid peptide production without TMV particle contamination. Another study used the hypertranslatable pEAQ vector, yielding peptides at a concentration of 0.07 mg/g of leaf tissues (Chaudhary *et al*., 2023), streamlining AMP production and showing potential for industrial‐scale application. Additionally, the pEAQ vector variants pHRE and pHREAC are more robust and provide additional vector choices for large‐scale production of AMPs (Peyret *et al*., 2019).

### Plant‐based expression platforms for AMP production

Plants offer a diverse platform to produce AMPs (peptides and proteins) using both transient and transgenic (nuclear or plastid) approaches for heterologous expression and large‐scale production (Chaudhary *et al*., 2023; Ghidey *et al*., 2020; Hoelscher *et al*., 2022; Lee *et al*., 2011; Łojewska *et al*., 2020; Martens and Demain, 2017; Paskevicius *et al*., 2017; Shanmugaraj *et al*., 2021; Yevtushenko and Misra, 2007; Zeitler *et al*., 2013). Nuclear and plastid transformation facilitate stable transgene expression. However, plastid transformation offers advantages such as exclusion from pollen transmission and prevention of epigenetic transgene silencing (Bock, 2015).

The advancement in molecular farming has also led to the development of plant cell suspension cultures, extendable to include other clonally propagated platforms in containment, including plant organ cultures like hairy roots, which have been explored for AMP production (Chahardoli *et al*., 2018; Hashemi *et al*., 2022; Khademi *et al*., 2019; Shams *et al*., 2019). Most importantly, the platform can integrate good manufacturing practices from the pharmaceutical industry and is scalable for large‐scale production.

### Mitigating toxicity to the plant host

One of the most significant concerns in expressing AMPs in plants is that AMPs might damage the plant's cellular membrane. Against this backdrop, NOMAD Biosciences have developed an approach utilizing inducible promoters, which involves spraying the necessary components onto the plants, triggering the controlled and efficient expression of peptides in the plants only when required, addressing concerns about potential deleterious effects of constitutive AMP expression on plant cells (Werner *et al*., 2011). Besides, sequestering AMPs into the inert compartment of plant cells offers another strategy to counteract their toxicity, as demonstrated by directing the antifungal protein AfpB to the apoplast, reducing toxicity and enhancing yield without requiring the expression of cell wall maintenance accessory enzymes (Shi *et al*., 2019). Furthermore, AMP expression can also be transcriptionally confined to rice endosperm, facilitating their high‐level accumulation without the necessity of complex DSP steps. In this context, Nadal *et al*. (2012) developed a strategy to mitigate AMP toxicity by expressing the BP100 peptide as tandem repeats. It was speculated that transgenic plants expressing this peptide have adequate agronomic performance and resistant phenotypes, attributed to a complex equilibrium between BP100der toxicity to plant cells, antimicrobial activity and the stress response induced by the transgene (Nadal *et al*., 2012).

Masking the AMP can also limit toxicity to the plant. For example, a recent study expressed AMPs tagged with a small ubiquitin‐like modifier (SUMO) domain, effectively masking their toxicity in plants, whether expressed in the plastids (Hoelscher *et al*., 2022) or the cytosol (Chaudhary *et al*., 2023). Fusing AMPs to larger proteins may make them less susceptible to protease digestion (Scotti *et al*., 2015), thus enhancing their stability (Hoelscher *et al*., 2022).

### Purification of plant‐produced AMPs

The purification process for peptides and proteins derived from plants typically follows a similar sequence involving expression and subsequent purification steps. The whole virus approach, specifically utilizing TMV, has proven successful in the production of short peptides. In this approach, the peptide is fused to the viral coat protein, and viral RNA is subsequently applied to the leaf surface by gentle rubbing with carborundum. The AMP‐conjugated TMV particles are then purified, and biologically active peptides are released through subsequent chemical cleavage using cyanogen bromide.

Both peptides and proteins can be easily accommodated in a de‐constructed vector and can be purified to the homogenous yield. For deconstructed viral vectors, *A. tumefaciens* is used to deliver DNA cassettes encoding AMPs into plant cells (agroinfiltration). It is at the user's discretion to express the AMPs in a selective plant host. AMPs can be produced in whole plants; calli; lab‐produced suspension cells; *Agrobacterium*‐induced plant hairy roots, seeds of rice, wheat and barley; leaves of tobacco, lettuce, cabbages and spinach and in tubers, rhizomes and bulbs. Tobacco plants, particularly *Nicotiana benthamiana*, have emerged as a major host for AMP production due to their compatibility with the requirements and therefore have been used in numerous industrial applications. Rice and spinach have also been used as hosts for AMP production by a few industries, but regulatory pressures arose due to concerns about the potential risks associated with genetically modified edible plants that produce pharmaceuticals. The selection of a plant host for industrial production of AMPs requires consideration of expression levels, genetic compatibility, protein folding, post‐translational modifications, biosafety, cultivation conditions, economic viability and regulatory compliance.

Plant platform additionally provides the flexibility to sequester AMPs to specialized compartments within plant cells for the purpose of maximizing accumulation levels. Besides, the use of specific domains such as SUMO or chitin‐binding domain (CBD) can be used by fusing them to the sequence to limit toxicity, and AMPs can be selectively pulled out from the pool of host proteins using affinity tags at either the N or C terminus, which can be selectively bound to resins or matrices. Once released from the plant tissue, AMPs can be purified through high‐performance liquid chromatography techniques and protease cleavage can be used to remove fusion sequences from the AMP. For example, engineered proteases such as SUMO can precisely cleave their recognition domain, removing it and the affinity purification tags from the peptides, leaving no residual amino acids. Finally, the peptides undergo polishing through size exclusion using peptide columns with organic modifiers like acetonitrile and hydrochloric acid (HCl), and then reverse‐phase chromatography to remove any remaining contaminants, including salt. The peptides are obtained in chloride salt form (paired with HCl), as opposed to the conventional trifluoroacetic acid salt, which is toxic to mammalian cells (Cornish *et al*., 1999).

Nomad Bioscience has successfully developed a simple acidic extraction protocol for industrial‐scale purification of antimicrobials from *N. benthamiana* plant slurry [GRN 593], bypassing the use of affinity columns, though still utilizing reusable packed (ion‐exchange) columns, which adds additional costs for cleaning and re‐validation of columns. To further address the cost issue, several research groups have explored the development of alternative purification protocols, the most interesting being the use of inert elastin‐like polypeptides (Floss *et al*., 2008, 2009) that reversibly change solubility at different temperatures, thus allowing simple, non‐chromatographic purification of AMPs and streamlining development and production; however, to date, only a few of these are approved for clinical translation. In addition, a generic purification protocol involving the use of ultrafiltration/defiltration to purify molecules less than 50 kDa (Opdensteinen *et al*., 2018) has been adopted as an intriguing alternative for industrial‐based processes. These require care to ensure that hydrophobic peptides do not adsorb to the filter matrices through ionic and hydrophobic interactions.

An alternative option in some cases is to use plant biomass without performing complete purification. For example, Ventria Bioscience (Sacramento, CA, USA) produced the antimicrobial proteins lactoferrin and lysozyme in rice endosperm, enabling its storage and transportation as dry seeds. This eliminates the need for a cold chain as the antimicrobials can be prepared in a local facility (Huang *et al*., 2002; Nandi *et al*., 2005).

Alternatively, rhizosecretion can produce therapeutically active proteins (Madeira *et al*., 2016a, 2016b). Apart from reducing contamination with host cell proteins and particles, this approach is suitable for application in plants growing hydroponically, is amenable to the addition of exogenous co‐factors to catalyse enzyme‐mediated post‐translational modification and can be harnessed to produce biologics, including AMP.

**Figure 3:**
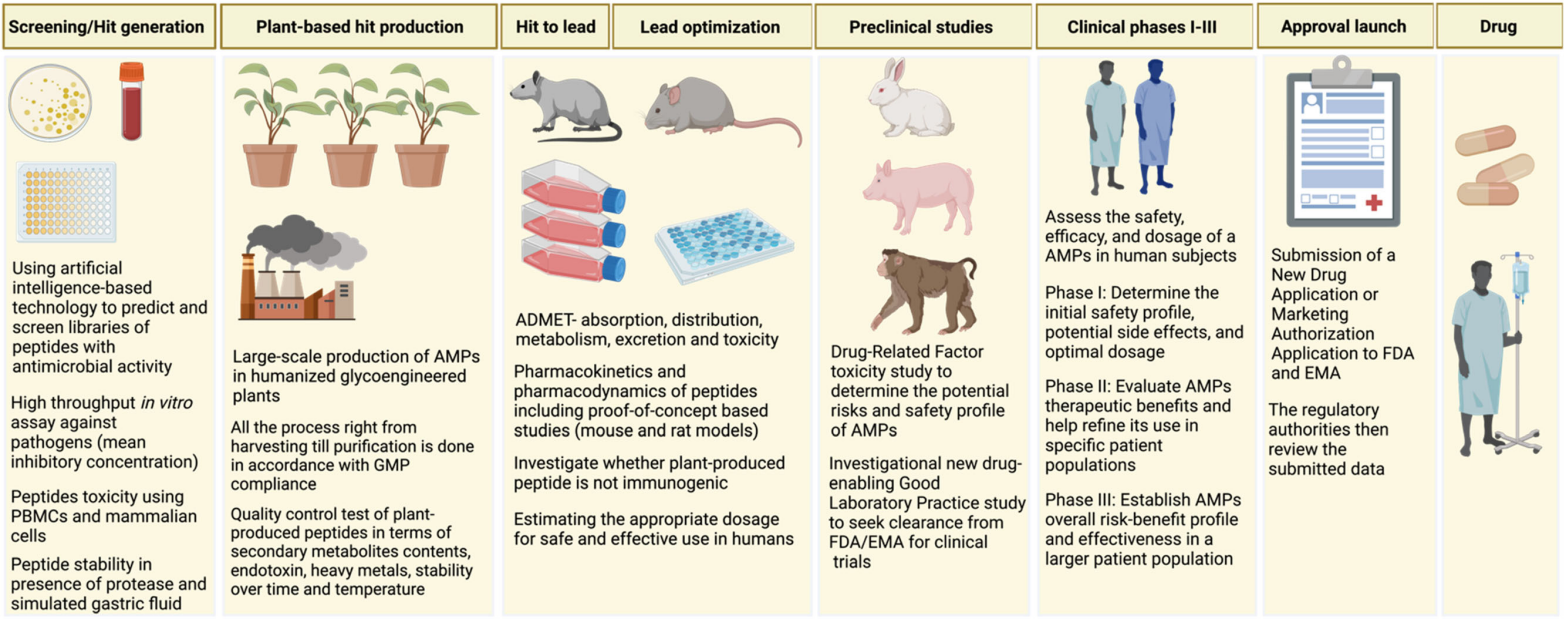
Harnessing the potential of the plant chassis: a comprehensive schematic of molecular farming methods to produce AMPs intended for clinical studies and the various stages involved in the approval process for plant‐produced peptide. EMA, European Medicines Agency.

As an alternative to using whole plants, suspension culture of plant cells such as tobacco (*N. tabacum* cv. Bright Yellow‐2) BY‐2 cells and carrot (*Daucus carota*) cultures are closest to traditional cell‐based platforms in the context of GMP manufacturing, and were the pioneer systems used in the development of molecular farming‐based clinical therapeutics (Lomonossoff and D'Aoust, 2016). Crude extracts of plant cell pellets can be applied topically (Lobato Gomez *et al*., 2021) and hold significant promise for drugs like AMP, which are limited to topical application and offer high prospects for cost‐effective treatment of surface wounds, particularly in developing countries. Overall, molecular farming has demonstrated significant potential for the industrial‐scale production of peptides that are now advancing into preclinical and clinical studies (Figure 3), with the goal of establishing a market niche.

### Improving AMP function by incorporating a chitin‐binding domain

Incorporating a chitin‐binding domain (CBD) in the peptide may help peptides reach target cells, thereby increasing the interaction between the peptide and cell membrane. For example, Dermaseptin B1 (DrsB1), a 31‐amino acid α‐helical cationic peptide from *Phyllomedusa* frogs, has strong activity against bacterial and fungal pathogens (Mor *et al*., 1994; Mor and Nicolas, 1994). When produced as tagged peptide with CBDs from the *Cladosporium fulvum* Avr4 effector protein and rice chitinases, this led to stable accumulation of peptide in tobacco plants. Production of another peptide, alfAFP (an AMP from *Medicago sativa*) fused with CBDs, similarly led to the stable production of this peptide and provided enhanced resistance against a broad spectrum of pathogens. Scanning electron microscopy of pathogens exposed to the recombinant peptides revealed considerable peptide‐induced rupturing and disintegration of cells (Badrhadad *et al*., 2018; Khademi *et al*., 2019, 2020; Nazari *et al*., 2018; Shams *et al*., 2019). Indeed, the heterologous expression of AMPs within plant hosts facilitates the production of AMPs for therapeutic applications (Neundorf *et al*., 2009).

### Engineering production of amidated peptides in plants

To date, clinical regimens for AMPs have been limited to topical application (Hakansson *et al*., 2019) or utilization as antibacterial agents in food (Magana *et al*., 2020). For such peptides to be used as systemic agents, they need to be modified, for instance by C‐terminal amidation (Erik Strandberg *et al*., 2007), to improve potency (Mor *et al*., 1994) and prevent enzymatic degradation (Moore *et al*., 1994). However, although significant parts of the post‐translational machinery are conserved between plants and mammals, plants lack enzymes that amidate proteins at the same positions as in mammals. In addition, the majority of clinical‐grade peptides are highly positively charged (Hancock and Sahl, 2006), and plant cells favour the expression of negatively charged peptides over positively charged ones, as native in planta peptides are generally less positively charged (Ghidey *et al*., 2020).

**Figure 4:**
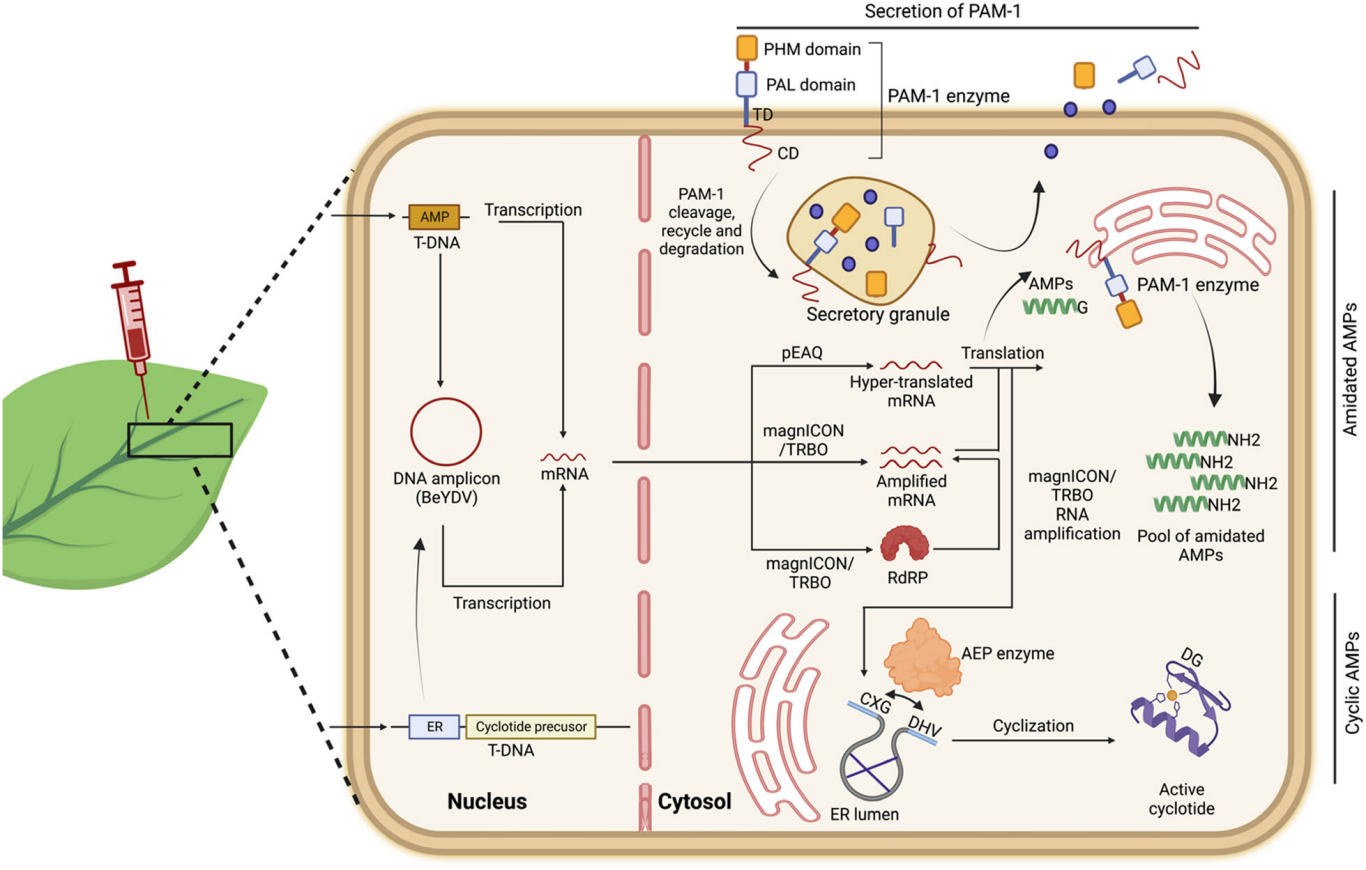
Engineering the production of amidated peptides in plant cells. *Agrobacterium tumefaciens* is used to transfer DNA encoding AMP expression cassette to the nucleus of the transgenic plant cell expressing peptidylglycine α‐amidating mono‐oxygenase (PAM). Following this, glycine‐extended peptide mRNAs are transcribed as single or multiple transcripts, depending on the expression cassettes used. Geminiviral vector (BeYDV‐based) vectors undergo DNA amplification through rolling‐circle replication within the nucleus before transcription. Once transcripts leave the nucleus, they can be directly translated into proteins using pEAQ or BeYDV, or they can first undergo an amplification step (using, e.g., magnICON or TRBO) with the help of RNA‐dependent RNA polymerase (RdRP) and then be translated. Once translated, the glycine‐extended AMPs are amidated by PAM. For production of cyclic AMPs (cyclotides), AMPs are expressed as cyclotide precursors bearing an N‐terminal CXG motif and a C‐terminal DHV, which when they are co‐infiltrated with asparaginyl endopeptidases, leads to ligation of the two termini and cyclization of the peptides. PAM, peptidylglycine α‐amidating mono‐oxygenase; PHM, peptidylglycine α‐hydroxylating monooxygenase; PAL, peptidyl‐α‐hydroxyglycine α‐amidating lyase; TD, transmembrane domain; CD, cytoplasmic domain; ER, endoplasmic reticulum; BeYDV, bean yellow dwarf virus; pEAQ, ‘easy and quick’ plasmid vector; AEP, asparaginyl endopeptidases.

In an attempt to achieve in planta production of amidated, hydrophobic and positively charged peptides with a favourable plasma half‐life, we recently succeeded in engineering a genetic circuit (Chaudhary *et al*., 2023). This approach involves the fusion of a SUMO domain to AMPs (Hoelscher *et al*., 2022) to mask their toxicity while enhancing their stability and solubility. A terminal glycine residue was appended to the C terminus of peptide, when expressed in a transgenic plant expressing the mammalian enzyme peptidylglycine α‐amidating mono‐oxygenase (PAM), not only resulted in higher level of peptide accumulation but also served as a substrate to enable post‐translational amidation of the peptides (Figure 4). These finding demonstrate a pioneering approach towards the development of post‐translationally modified peptides in plants, opening up new avenues for the production of modified peptides via molecular farming.

### Engineering production of cyclic peptides in plants

Experimental studies have demonstrated that cyclic derivatives of peptides exhibit enhanced bioactivity due to their structural rigidity, improved selectivity and notably increased stability against digestive enzymes (Raghuwanshi *et al*., 2017; Wang *et al*., 2019; Yap *et al*., 2014). This enhanced stability significantly impacts the oral absorption and bioavailability of peptides in the gut (Kong *et al*., 2020). Cyclic peptides are mainly synthesized using the CyClick chemistry (Adebomi *et al*., 2019); however, this method often causes production problems in peptide synthesis due to the formation of undesired by‐products such as linear and cyclic dimerization or oligomerization, reducing the overall yield.

To produce cyclic peptides in plants, researchers have co‐expressed cyclotide precursors bearing an N‐terminal propeptide (NTPP) motif and a C‐terminal propeptide (CTPP), with asparaginyl endopeptidases (AEPs) sourced from cyclotide‐producing species like *Oldenlandia affinis* and *Clitoria ternatea* in *N. benthamiana* (Poon *et al*., 2018). Initially, the AEP enzyme catalyses cleavage at the NTPP and CTPP sites, followed by a final cyclization reaction resulting in the formation of peptide bond linking the first glycine residue and the last asparagine residue.

This method has significantly increased the yields of cyclic peptides within the plant, making it a promising avenue for developing efficient and versatile biofactories capable of producing therapeutic cyclic peptides (Figure 4). In fact, Kalata B1, a cyclic AMP, has recently been produced in an *N. benthamiana* expression system (Jackson *et al*., 2023), and these results could be extended to enable production in edible plants such as spinach and lettuce. Synthetic Kalata B1, produced using SPPS, when administered orally, can ameliorate symptoms of multiple sclerosis in a mouse model (Thell *et al*., 2016). Although the efficacy of plant‐produced cyclic AMPs still needs to be validated in preclinical mouse studies, the successful engineering of complex cyclotide structures in plants has opened avenues for the plant‐based production of cyclic peptides.

## Plant molecular farming for sustainable AMP production

The field of AMPs has often been deprioritized for peptide drug development, as many candidate peptide drugs have failed to meet their secondary endpoints, particularly those related to reduced toxicity (Hancock and Sahl, 2006). This is because peptides derived from organisms or tissues are generally simply tested for activity in bacterial cultures and then moved too rapidly into preclinical and clinical testing without being fully optimized (Fox, 2013). However, lead peptides that are tested in animal models often still fail to advance into clinical studies, primarily due to manufacturing difficulties, as observed in the case of plectasin NZ2114 and many other lead peptides (Fox, 2013).

In addition, the growing number of novel peptide sequences available for preclinical testing has led to a demand for more scalable and cost‐effective production methods in the manufacturing industry. This demand has been further heightened by the emergence of an AI platform (Das *et al*., 2021; Ma *et al*., 2022; Torres *et al*., 2022b; Wong *et al*., 2023), which has underscored the need for efficient production methods to keep pace with the expanding database of peptide sequences. To date, most of the AMP that have been approved by FDA remain in production, with SPPS being the primary manufacturing method, followed by recombinant production in bacteria or yeast. Although SPPS and recombinant systems have been successful for producing AMPs, using plants as self‐contained disposable bioreactors is a promising alternative that provides a ‘greener’ solution, mitigating potentially harmful consequences to the environment.

### Reduced investment and cost

For the past three decades, the most widely used method for recombinant production of peptides or other biologics has been the culture of cells in stainless steel bioreactors, a costly proposition. The cost of stainless steel has tended to increase over time, which directly affects the expenses associated with traditional bioreactors (Buyel, 2018). Ferreira *et al*. (2018) estimated that the operational expenses (OPEX) and cost of goods sold (COGS) for a 280 MT/year facility that produces cell wall degrading enzyme β‐glucosidase in *E. coli* cells, assuming an 80% working volume of the fermenter and a titre of 15 g/L, amount to $10.7 million per year and $37 kg^−1^ respectively (Ferreira *et al*., 2018). Additionally, running the fermenter incurs an electricity cost of $0.5 million, as well as costs associated with cleaning and maintenance, and increases the carbon dioxide footprint of the corresponding products (Buyel, 2018). For a similar transgenic plant‐based ‘green synthesis’ production facility, where the plant itself constitutes a self‐contained disposable bioreactor and gets completely disintegrated during the extraction process, e.g. using blade‐based homogenizers (Buyel and Fischer, 2014b; Hassan *et al*., 2014), the estimated OPEX is $20 million per year, with a COGS of $9 kg^−1^, assuming an expression level of 4 g/kg fresh weight (Ferreira *et al*., 2018; Tusé *et al*., 2014). This translates to a cost reduction of >50% compared to the *E. coli* facility, along with a significant reduction in the requirement for cleaning and validation procedures, achieving cost savings of more than $10 million at the production scale along with a reduced carbon footprint that makes it more sustainable (Buyel, 2018). Generally, the cost of producing proteins in plants is estimated to be 10–50 times lower than that of *E. coli* fermentation (Kusnadi *et al*., 1997).

We obtained similar results in a recent study: we estimated the cost of producing plant‐manufactured AMPs to be ~$74 g^−1^ (equivalent to $1 per dose), with a 50% reduction in OPEX compared to bioreactor production (Chaudhary *et al*., 2023). This analysis is based on a techno‐economic assessment (TEA) model assuming that the upstream facility is constructed with cutting‐edge technologies such as LED lighting, vertical farming, hydroponics and vacuum‐based infiltration, which are predicted to become increasingly affordable and enable higher biomass concentrations without increasing costs (Chaudhary *et al*., 2023). Overall, TEAs predict substantial decreases in capital expenditures and >50% reductions in the cost of producing goods through utilizing plants as a platform compared to other methods. However, despite the direct relevance of these simulations, it will still be necessary to verify these theoretical predictions by assessing large‐scale procedures. Furthermore, licensing fees such as those applicable to specialized mammalian production cell lines like CHO DG44 (Reinhart *et al*., 2015) might also apply to plant lines that provide humanized glycosylation or other post‐translational modifications (Jansing *et al*., 2019). Some AMPs such as diptericin, drosocin, formaecin, lebocin and phyrrorricin are synthesized as glycoconjugates with *O*‐linked glycans added post‐translationally, which are essential for peptide recognition, binding and protein–protein interactions (Wang, 2012). However, the theoretical risk associated with non‐mammalian glycans, previously regarded as the Achilles heel of molecular farming‐derived therapeutics, where glycan differences could introduce a pharmacokinetic and/or safety risk in systemic administration, has not materialized in practice (Tuse *et al*., 2020). Regardless, humanizing such AMPs via glycosylation is crucial, ensuring the peptide's clinical suitability for therapeutic purposes.

### Reduced DSP burden and associated cost

To increase the sustainability of a production unit, it is important to identify and address any added burden on the DSP, as this accounts for 80% of total production costs (Buyel, 2015; Wilken and Nikolov, 2012). Doing so can reduce the overall COGS, resulting in a more sustainable and profitable operation. One approach is to use an alternative method of protein recovery, involving centrifugal extraction (McCormick *et al*., 1999), screw‐pressing (Buyel and Fischer, 2014a) or treatment at a moderate temperature (~65°C) and low pH (~5.5) (Hassan *et al*., 2008). These approaches decrease the levels of host cell protein and reduce associated expenses by 75% (Buyel, 2018). McNulty *et al*. (2020) demonstrated this reduction in downstream costs through TEA modelling, showing that producing antimicrobial proteins in plants by this method has an OPEX of $1.43 million/year and a COGS of $3.00–6.88 g^−1^, which makes it a competitive option compared to traditional food safety treatments.

Interestingly, the calculations for plant‐based AMP production reveal an OPEX of $12.5 million/year and a COGS of $74 g^−1^, which translates to ~$1 per dose, but the calculated OPEX is notably higher compared to proteins. This discrepancy in cost is not associated with the number of amino acids added to the peptide backbone, but reflects the cost associated with the DSP to achieve the required purity and homogeneity for clinical applications, particularly for systemic injections. In contrast, the antimicrobial proteins discussed by McNulty et al. do not require such extensive purification, especially when used as antibacterial agents in food‐related applications.

It is interesting to note that even though multiple chromatography steps are involved in the production of plant‐based clinical peptides, the platform still requires significantly less capital investment and lower cost of goods, as compared to *E. coli* fermentation or mammalian culture (Chaudhary *et al*., 2023). The final COGS was estimated to be <$100 g^−1^, which is quite competitive when compared with chemical synthesis and *E. coli*‐produced peptides. This demonstrates that the production of plant‐based AMPs or proteins is significantly less costly despite the need for multiple chromatography steps (Chaudhary *et al*., 2023).

Nandi *et al*. (2005) demonstrated that DSP is negatively correlated with production capacity. An analysis of the cost of DSP for producing another antibacterial protein rhLF in stable transgenic rice seed showed an expression level of 0.5% rhLF, a production capacity of 600 kg purified rhLF (>90% purity) and a cost estimated at $5.90 g^−1^. However, reducing the expression level to 0.005% rhLF in rice flour and the production capacity to 200 kg/year increased the estimated cost of DSP by 40%, to $375 g^−1^ (Nandi *et al*., 2005). The study demonstrates that the expression level of proteins in plants significantly influences processing cost.

To avoid extraction costs completely, the rhizosecretion platform (Madeira *et al*., 2016a, 2016b) has been used to produce pharmaceuticals from transgenic plants grown hydroponically in vitro; however, its successful implementation requires a thorough evaluation of the total costs involved in DSP and the overall COGS for the final product, which can be done through TEA. It is also important to note that this approach is restricted to transgenic plants and products that can tolerate the conditions of the hydroponic solution when secreted. In addition, using this method may lead to regulatory scrutiny of the product due to the non‐sterile and potentially poorly defined conditions involved (Buyel, 2018).

### Cost per dose

The definition of successful peptide drug implementation should shift from cost per dose to cost per dose delivered to precisely estimate the cost of introducing a new peptide (Muttenthaler *et al*., 2021; Ngabo *et al*., 2015). This implies that the total cost of peptides encompasses various expenses beyond production, including distribution and storage costs. Plants offer a promising avenue for producing peptides on a small scale and at a reduced cost, eliminating the need for cold chain storage and addressing peptide equal distribution, even in regions with limited access to cGMP‐level SPPS and bacterial culture systems. TEA models for biopharmaceutical manufacturing are rarely presented and discussed in detail, but analysis of a limited number of molecular farming‐based products has indicated that plant‐produced biopharmaceuticals can be economically advantageous compared to other platforms. The estimated costs of manufacturing an antimicrobial drug using a plant‐based platform range from $3.00 to $74 g^−1^ (Chaudhary *et al*., 2023; McNulty *et al*., 2020), which translates to ~$1 per dose. Moreover, the production costs of recombinant antimicrobials (Alam *et al*., 2018; McNulty *et al*., 2020; Nandi *et al*., 2005), enzymes (Corbin *et al*., 2020) and antibodies (Nandi *et al*., 2016) utilizing plant‐based methodologies are expected to be comparable to, or even less expensive than, mammalian‐cell‐based production costs. A recently introduced simplified TEA model for mAb production using various platforms can also be applied to other production platforms, by considering universal factors that impact the cost and efficiency of bulk drug manufacturing. Although these costs are theoretical estimates from in silico simulation, they are highly relevant, but it is still crucial to validate them on a large‐scale platform.

### Resource‐full, biodegradable biomass and reduced environmental burden

**Figure 5:**
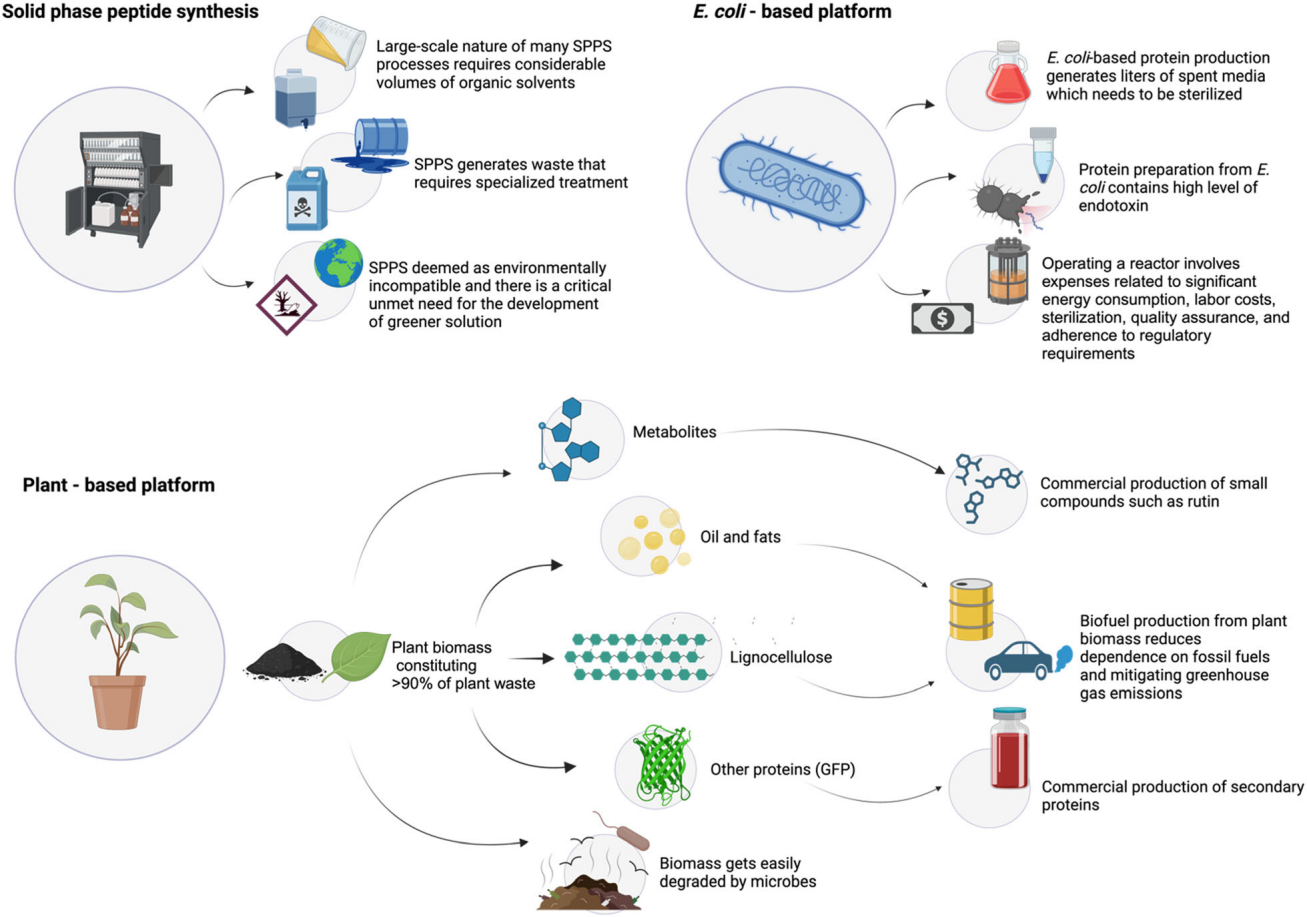
Plants as a chassis for sustainable biomanufacturing. Mammalian cell culture‐based processes relying on single‐use equipment and plant‐based processes both produce waste. Plant molecular farming typically yields a primary product comprising less than 1% of the total biomass produced in the initial stages of processing. However, the remaining biomass can be harnessed to produce additional value‐added products. These side streams have the potential to significantly enhance the economic value of the plant‐based process, potentially doubling revenue.

In the molecular farming platform, the target protein constitutes only <10% of the total soluble protein, even when expression levels exceed 2 g/kg of biomass (Buyel, 2018). The remaining fraction comprises 90% of biomass (Figure 5), which translates to a significant economic potential amounting to millions of dollars, as the unused fraction sometimes consists of fluorescent marker proteins (priced at €6250–6360 per 5 mg DsRed, for example; Buyel, 2018), lipids and lignocellulose (which can be processed to obtain additional chemical building blocks or biofuels) and secondary metabolites such as rutin (valued at €2.18 g^−1^). In a case study (Buyel, 2018), integrating the DsRed or rutin purification process with the leftover biomass (assuming an expression level of 0.7–1.0 g/kg of biomass) can result in an additional revenue of €0.84 or €1000 kg^−1^ biomass respectively.

The sustainability of plant‐based production is further reinforced by the fact that any plant material left over after extraction will rapidly biodegrade under typical environmental conditions (Baldrian, 2017; Miki *et al*., 2010; Zhu *et al*., 2013). This further reduces the volume of waste and the requirement for costly and specialized disposal facilities, which are necessary for other proteins production platforms (Buyel, 2018). For instance, Kopach (Eli Lilly and Company, Senior Advisor) pointed out a major sustainability issue with SPPS, indicating that producing 1 kg of peptide results in generating between 3000 and 15 000 kg of hazardous waste (Kopach, 2018). Additionally, the expense associated with disposing of toxic waste in the peptide manufacturing sector is approximately between $1 billion and $1.5 billion, calculated in relation to the yearly revenue generated from therapeutic peptides, which surpasses $20 billion (Bukrinski, 2020) as stated by Dr. Jens Bukrinski, who served as the former Head of Research and Development at Novo Nordisk and Novozymes Biopharma. These waste predominantly contains dimethylformamide (DMF), dichloromethane (DCM) and *N*‐methyl‐2‐pyrrolidone (NMP), all of which are suspected to be aggressively harmful and are under consideration for restriction by the European Chemicals Agency's (ECHA) Registration, Evaluation, Authorization, and Restriction of Chemicals (REACH) regulations (Martin *et al*., 2021).

Bacterial platform produces a significant quantity of endotoxin, which is extremely pathogenic and thus requires specific handling before disposal (Figure 5). Conversely, contaminants resulting from plant molecular farming are less harmful in comparison to endotoxins produced during bacterial expression (Merlin *et al*., 2014). Besides, plant‐based processes minimize liquid waste, do not necessitate specific handling and require heat treatment for only a subset of processes, such as those relying on transient expression facilitated by *A. tumefaciens* infiltration.

## Patents, clinical translation and FDA approval

While plant‐based production of AMPs is still in the research and development phase, and only a limited number of patent applications for AMPs are currently in progress, there is already an existing list of patents for plant‐produced antimicrobial proteins. It is expected that a similar standard procedure will be followed for the patenting and clinical translation of peptides in the future.

Plant‐based antimicrobials and recombinant therapeutics necessitate strict adherence to cGMP guidelines for successful commercialization. Nomad Bioscience GmbH (Germany) utilizes the proprietary magnICON technology platform (U.S. patent US20130212739A1) for plant‐based protein expression. They achieved the first Generally Recognized as Safe (GRAS) status in the United States in 2015 for colicins derived from plants, specifically spinach, red beet or lettuce leaves [GRN 593]. These recombinant colicins, expressed using TMV or potato virus X, can be applied as antimicrobial agents to vegetables, fruits or meat products at recommended levels of 0.5–5 mg/lb [GRN 775]. In preclinical studies, plant‐produced colicin cocktails reduced bacterial load by 1–3 logs when applied to various food matrices. Moreover, the antimicrobials have demonstrated low toxicity in in vitro studies on human and primate cells, with low developmental potential for allergenicity or immunogenicity, emphasizing their favourable safety profile [GRN 593].

Since 2015, NOMAD Biosciences has acquired a significant number of patents, with several of their plant‐based antimicrobial products showing positive outcomes in preclinical/clinical studies, and a subset of these products have received ‘No Questions’ letters from FDA [GRN 676, GRN 775, GRN 802, GRN 593], positioning them for market delivery and commercialization (https://www.nomadbioscience.com). Nomad's lead plant‐produced antimicrobial candidates colicins, salmocins and endolysins, produced in a current GMP (cGMP)‐compliant manufacturing facility, have successfully entered clinical trials, whose preliminary results have shown promising outcomes (https://www.nomadbioscience.com). While most of Nomad's lead peptides are being developed primarily as food antimicrobials, others have been produced and successfully employed in clinical trials to target drug‐resistant bacteria and respiratory viruses (https://www.nomadbioscience.com).

In contrast to plant‐produced antimicrobials, Divergicin M35, which is produced by *Carnobacterium divergens* M35, obtained regulatory approval in Canada in 2016 (Canada Health Department approval reference number: NOM/ADM‐0079). However, biocontainment is the main concern raised in using a bacterial chassis to produce the antimicrobials [GRN 762], whereas plant chassis, because of their immunologically inert nature, did not interfere in the regulatory process and antimicrobials produced in these systems swiftly received GRAS status.

The convergence of lower capital costs, reduced production expenses and increased acceptance of plant‐based production methods has facilitated the development of second‐generation ‘biobetter’ antimicrobial therapies. We have also established plant‐based peptide production in our greenhouse facility located at the King Abdullah University of Science and Technology in Saudi Arabia; a U.S. patent application (63/484 445) is currently pending for this technology, and preclinical studies on a murine model of infection are currently underway. Grams of purified peptides, enough to supply a patient for a lifetime, can be produced at a cost as low as $74 (~$1 per dose), which is significantly cheaper than those produced with conventional expression systems, which are estimated to cost around a hundred times more.

## Regulatory guidelines

As a general rule, the more confined and controlled the manufacturing process is spanning from harvesting to final production, the smoother it will be to channel into the drug pipeline and the further marketing approval; the detailed framework has been described elsewhere (EMA, 2006; FDA, 2002). Also universally, *Nicotiana*‐derived products have shown high levels of safety in clinical studies, even when purity specifications were set at ≥90% (Tuse, 2011); however, it is still essential to prepare safety documentation in compliance with regulatory standards.

Upstream processes involving growing whole plants indoors under controlled conditions, including plant cell culture methods, followed by downstream purification carried under GMP compliance, have been fared as best when scrutinized by regulatory affairs. This holds true for non‐food plants used as expression hosts, especially *Nicotiana* species. However, in the context of field‐based production, it is still feasible in the United States, but such methods necessitate further permits and scrutiny from the United States Department of Agriculture (USDA). In May 2020, the USDA Agricultural Plant Health Inspection Service (APHIS) revised regulations governing the interstate transportation or release of genetically modified organisms (GMOs) into the environment, aiming to promote innovation and decrease the regulatory burden on the industry. This revision, known as the ‘SECURE Rule revision of 7 Code of Federal Regulations (CFR) 340’, sought to enhance the precision of regulating such practices (USDA/APHIS SECURE Rule revision to 7 CFR 340. accessed 4 February 2024). In Europe, the manufacturing of molecular farming‐based products using GMOs or transient expression in open field is subjected to rigorous regulatory scrutiny in the European Union (EU), and several statues have been established to mitigate potential environmental, food and public risks associated with these processes.

In Europe, biologics, regardless of the manufacturing platform used, are regulated by the European Medicines Agency (EMA), and in the United Kingdom, by the Medicines and Healthcare products Regulatory Agency. Just like other biotechnology‐derived drugs, antimicrobials derived from genetically modified plants must comply with the regulations outlined by the European Commission (EC), including Directive 2001/83/EC and Regulation No 726/2004. Furthermore, it is crucial that all stages of the biologics production process, from growing plants in greenhouses (regulated by Directive 2009/41/EC) to harvesting of the biologics to the final purification stage, comply with additional statutes (only for GM plants regulated by EC under Directive 2001/18/EC and 1829/2003/EC) and stringently adhere to current cGMP standards.

Although many plant antimicrobials are generally recognized as safe (GRAS) for specific food applications, their use in other food applications is not explicitly approved. For AMP‐containing preparation to be used as food preservatives, compliance with the specifications outlined in Regulation 1333/2008/EC is necessary. Phenols are the primary contaminants in plant‐based purification processes, and notably, they are not included in the positive list of approved food preservatives. Approval from regulatory authorities for the use of plant extracts as food additives is crucial to guarantee consumer safety and confidence in food products. Such authorization is evaluated by qualified experts and can be approved by the FDA based on scientific evidence and expert consensus (FDA, 2018). In the toxicological evaluation, the compound's concern level (low, intermediate or high) is determined based on its predicted toxicological potential from its chemical structure and the estimation of cumulative human exposure. Compounds with a low concern level in toxicological evaluation may require only genetic and short‐term toxicity tests in rodents through peer‐reviewed, evidence‐based publications, whereas compounds with a high concern level necessitate 1‐year toxicity studies with non‐rodents, chronic toxicity assessments, carcinogenicity studies with rodents and possibly human studies (FDA, 2006).

## Overcoming barriers

Although it has been proven in numerous instances that plants have excellent potential to produce AMPs of high quality, only a few plant‐derived AMPs have advanced to clinical trials, and even fewer have successfully reached the market to date. The primary reasons include inadequate yields, high purification costs pertaining to DSP and the challenges posed by regulatory compliance. The integration of molecular farming with synthetic biology has led to the development of robust vectors, enabling production of therapeutics in milligram quantities within a few days following the delivery of DNA constructs but also pave the way for novel applications, particularly in the context of oral delivery using plastomic (Khan and Daniell, 2021; Singh et al., 2021) or seed‐based expression systems (Morandini et al., 2011).

According to the FDA Critical Path Report, DSP emerged as a significant factor contributing to the consistent failure of plant‐derived proteins to progress into clinical or industrial settings (Lico *et al*., 2008). However, novel approaches are being developed which include chemical‐based approaches like enzymatic hydrolysis, non‐chemical methods such as ultrasound, microwave, pulse electric fields and high‐voltage electric discharge, as well as solvent‐based techniques like subcritical and supercritical fluids, and hydrotropic and deep eutectic solvents, all aimed at improving the DSP of plant materials (Gençdağ *et al*., 2021).

According to James M Utterback (1975) model, developing an intellectual property right for an innovative plant‐based expression system and owning it is seen as constructive for translating it into an industrial setting. However, relying solely on individual proprietary technologies can impose restrictions on industry partners, limiting their freedom to explore alternative approaches and impeding collaboration. This constraint can hinder engagement with experts from research organizations specializing in AMPs, other industries and government regulators. It is becoming essential to establish a commonly unified regulatory framework to also facilitate the development of molecular farming‐based antimicrobial platforms in developing countries. Moreover, it is crucial to enhance public awareness regarding plant‐based production and its safety for various reasons, such as fostering acceptance among the general population and receiving (and acting on) feedback.

## Future outlook

In terms of producing AMPs, SPPS, bacterial and yeast systems have already established a niche in the market for large‐scale production, and it is therefore may be challenging for plant‐based systems to gain a market foothold. To shift the balance in favour of plants, it will be essential to continue harnessing the untapped potential of plants to promote sustainable plant‐based AMP production. Judging from the number of patents issued, successful completion of current and soon‐to‐be‐implemented clinical trials, and FDA approval for plant‐based antimicrobials, there is compelling evidence that these products have met the necessary criteria in terms of clinical effectiveness, industrial viability and market acceptance. In addition, the cost of plant‐derived AMPs can be as low as $1 per dose, increasing their competitiveness as compared to AMPs produced in traditional hosts. Although the TEA models predict a significant reduction in capital investment and COGS of plant‐produced AMPs, these projections will need to be confirmed through industrial‐scale production of AMPs. Using plants as a means of sustainable production could prevent future penalties from regulatory agencies since this method aligns more effectively with the objectives of environmental protection laws. Furthermore, molecular farming could enable the production of AMPs in space, whether on a space station or during prolonged missions to destinations like Mars, addressing the challenges of pharmaceutical supply in extraterrestrial destinations. Thus, plant‐based platforms have the potential to facilitate rapid and widespread distribution of antimicrobial drugs, thereby fostering equitable access to pharmaceutical products on a global scale.

## Funding

This work was supported by BAS/1/1035‐01‐01 baseline and KAUST Smart Health Initiative funding to MM.

## Conflict of interest

The authors declare no competing financial interests.

## Data Availability

Data sharing is not applicable to this article as no new data were created or analyzed in this study.
